# Geometric Morphometrics of Rodent Sperm Head Shape

**DOI:** 10.1371/journal.pone.0080607

**Published:** 2013-11-28

**Authors:** María Varea Sánchez, Markus Bastir, Eduardo R. S. Roldan

**Affiliations:** 1 Reproductive Ecology and Biology Group, Museo Nacional de Ciencias Naturales (CSIC), Madrid, Spain; 2 Department of Paleobiology, Museo Nacional de Ciencias Naturales (CSIC), Madrid, Spain

## Abstract

Mammalian spermatozoa, particularly those of rodent species, are extremely complex cells and differ greatly in form and dimensions. Thus, characterization of sperm size and, particularly, sperm shape represents a major challenge. No consensus exists on a method to objectively assess size and shape of spermatozoa. In this study we apply the principles of geometric morphometrics to analyze rodent sperm head morphology and compare them with two traditional morphometry methods, that is, measurements of linear dimensions and dimensions-derived parameters calculated using formulae employed in sperm morphometry assessments. Our results show that geometric morphometrics clearly identifies shape differences among rodent spermatozoa. It is also capable of discriminating between size and shape and to analyze these two variables separately. Thus, it provides an accurate method to assess sperm head shape. Furthermore, it can identify which sperm morphology traits differ between species, such as the protrusion or retraction of the base of the head, the orientation and relative position of the site of flagellum insertion, the degree of curvature of the hook, and other distinct anatomical features and appendices. We envisage that the use of geometric morphometrics may have a major impact on future studies focused on the characterization of sperm head formation, diversity of sperm head shape among species (and underlying evolutionary forces), the effects of reprotoxicants on changes in cell shape, and phenotyping of genetically-modified individuals.

## Introduction

Sperm cells are very diverse in size and shape among taxa [1]–[3]. Evolution in size and shape of male gametes may be driven by two main selective forces: sperm competition [2], [4], [5] and female reproductive biology [6]–[9]. When a female copulates with more than one male during a reproductive period, sperm from rival males compete in the female tract to fertilize the ova. This evolutionary force favors ever more competitive ejaculates, improving several ejaculate traits that are important determinants of fertilization success [10]. Sperm competition has been associated with an increase in total sperm dimensions [11]–[14]. Longer sperm may be able to produce more energy in the midpiece [15] or the principal piece of the flagellum [16] and generate higher propelling thrust [13] and, as a consequence, swim faster [11], [12], [17]–[19]. A higher swimming speed may also be achieved if sperm have more hydrodynamically-efficient heads, which reduce drag [17]. Hydrodynamic efficiency may be achieved by modifications of the ratio head length/head width, resulting in a more elongated sperm head, and this may also be influenced by sperm competition [12]. Rodents exhibit the widest range of sperm sizes among eutherian mammals [2], [14]. They also show considerable differences in head shape morphs, from simple oval heads to falciform ones, with one or several apical hooks, or elongations in the base of the head [2], [20], [21].

Traditionally, sperm heads have been analyzed manually using one-dimensional measurements of length, width, and area [1], [22] which have gained in precision when computers and image analysis software were introduced [23]. In any case, accuracy of sperm morphometry depends on several factors [24]–[27], including potential variations between laboratories [28], [29]. To further improve sperm morphometry assessments, automated sperm morphometry analysis (ASMA) systems were developed [30]. They provide information on sperm head linear dimensions (i.e., size) and use a series of mathematical formulae to calculate dimensions-derived parameters (as an approximation to head shape). The method was originally designed for human sperm [31] and it has been adapted to several animal species (e.g., [32]–[35]). Fourier analysis is another computer-aided method that has been employed [36]–[38], but its use is less extended than ASMA. It is based on the use of a succession of points located by a coordinate system that fits the cell perimeter to a Fourier function. These techniques can identify more features of morphological variation in sperm than manual methods. However, none of them utilize the theoretical background of modern shape analysis that may enable one to distinguish between size and shape, nor does any of them allow for quantitative incorporation of specific biologically meaningful anatomical features. This is because localizations of anatomical structures are not captured by traditional measurements. Furthermore, traditional morphometry faces difficulties when attempting to measure spermatozoa from species with very elaborate head shapes, such as those of rodents, because they cannot capture the full complexity of sperm heads, so a more sophisticated approach is required.

Geometric morphometrics [39]–[43] may be potentially useful tools for quantification of sperm head morphology. This is because geometric morphometrics are based on landmarks, which specify the exact spatial position of a given anatomical structure. Geometric morphometrics methods are elaborated on the basis of a theory about shape [44], according to which the shape of landmark configurations is not affected when scaling, rotation or translation is applied to them. Thus, landmark configurations of the measured specimens are iteratively translated, rotated and rescaled (to a common size) with the advantage of disentangling shape from size, allowing for separate analyses of these traits.

Procrustes-based geometric morphometrics [39]–[43], [45] could thus be used to analyze the geometric properties of sperm heads addressing the spatial configurations of landmark coordinates. Information that is unrelated to the shape of the objects, such as absolute position, orientation and scale, is extracted during the Procrustes superimposition and the remaining shape variables, Procrustes residuals or other variables derived from thin plate spline (TPS) techniques (partial warps and uniform component scores), are analyzed by multivariate statistical procedures [40], [42], [43]. Thin plate splines can be used further to quantitatively visualize the results as smooth grid transformations between two landmark configurations which, besides the aforementioned quantification of spatial anatomical features, is the second key advantage of landmark geometric morphometrics, as this transformation provides clues to identifying anatomical features.

Geometric morphometrics have not been used before in comparative analyses of mammalian spermatozoa. A recent study of sperm head morphology of the house mouse (*Mus domesticus*) used geometric morphometrics principles in an attempt to assess if sperm competition influences sperm head morphology. However, the analyses focused mainly on sperm head "hookedness", and no relation was found between hook patterns and sperm competition [46], [47].

In the present study we explored whether geometric morphometrics is a more detailed and accurate approach to quantify size and shape differences in rodent sperm heads. To this end, we compared methods currently used in sperm morphometry (i.e., measurement of linear dimensions, and calculations of dimensions-derived parameters using various formulae) with results obtained using geometric morphometrics.

## Materials and Methods

### Ethics Statement

All animal handling was done following Spanish Animal Protection Regulation RD1201/2005, which conforms to European Union Regulation 2003/65. The research protocol was approved by the Ethics Committee of Spanish Research Council (CSIC). Animals were sacrificed by cervical dislocation, which is regarded as a humane method by European Union and Spanish regulations. None of the species included in this study is considered to be endangered or is included in the list of Spanish protected species (Spanish Order AAA/75/2012 of the Ministry of Agriculture, Food and Environment). Animals were captured with permissions from Junta de Castilla y León and Comunidad Autónoma de Madrid, Spain.

### Sperm Collection and Preparation

We examined spermatozoa from four species of rodents from natural populations of the Iberian peninsula: *Arvicola sapidus, Arvicola terrestris, Clethrionomys glareolus, Microtus arvalis.* Animals were captured during their reproductive season (April-June). After the animal dissection, caudae epididymides were cut and placed in 1 ml of modified Tyrode's medium containing Hepes buffer [48] at 37°C to allow sperm cells to swim out into the medium. Spermatozoa were smeared onto slides, fixed with formaldehyde in a phosphate buffer, and stained with Giemsa as previously described [10], [49] and examined using bright field microscopy. All samples were evaluated and photographed at 1000x magnification for subsequent digitalization using an Eclipse E-600 microscope (Nikon, Tokyo, Japan) with Pan-Fluor optics and a DS5 camera (Nikon, Tokyo, Japan). Spermatozoa were photographed by using the software NIS-Elements (Nikon, Tokyo, Japan). Each individual contributed with 25 different measurements to the sample. Thus, there is not pseudoreplication in our data set.

### Sperm Measurements

Linear dimensions were obtained by measuring captured sperm images using ImageJ software v.1.41 (National Institutes of Health, Bethesda, MD, USA) [49]. Measures included head length (HL), head width (HW), head area (A) and sperm head perimeter (P).

### Sperm Analysis Using Geometric Morphometrics

Geometric morphometrics have the advantage, over other morphometric methods currently in use, of dissociating the size and shape of an object and analyzing both of them separately. The method currently used for geometric morphometrics analysis is the generalized least squares Procrustes superimposition method [41], [43]. This method transforms the raw data through rotation, scaling and translation to remove all information unrelated to shape and minimizes differences between landmark configurations and the Procrustes distance [40]. The latter is the distance between two landmark configurations in Kendall's shape space, and corresponds to the square root of the summed squared distances between homologous landmarks in the space of the landmark configurations. A landmark is a point in a bi- or three-dimensional space that corresponds to the position of a particular trait in an object. Landmarks are classified into three types [40]. Landmarks type I are defined by particular structures, such as tissue boundaries, bone sutures or other, anatomically identifiable structures. Landmarks type II are defined as points of maximum and minimum curvature. Landmarks type III are defined geometrically, and they only can be identified in relation to the axes of the entire structure [43]. The use of many type III landmarks (or semilandmarks) allows for a quantification of curved morphological structures for analysis within a geometric morphometrics framework when no type I or type II landmarks are available [40], [50]. Semilandmarks require a specific processing (resliding) because only a limited part, which informs about curvature, is biologically meaningful [50]. Their eventual position along the curve is then determined such that it minimizes bending energy between specimens in relation to the type I and II landmarks. Two principal approaches to sliding of semilandmarks are currently described [43]. One of these is the minimization of bending energy [51], [52], while the other is minimization of Procrustes distance from the mean shape [43].

**Figure 1:**
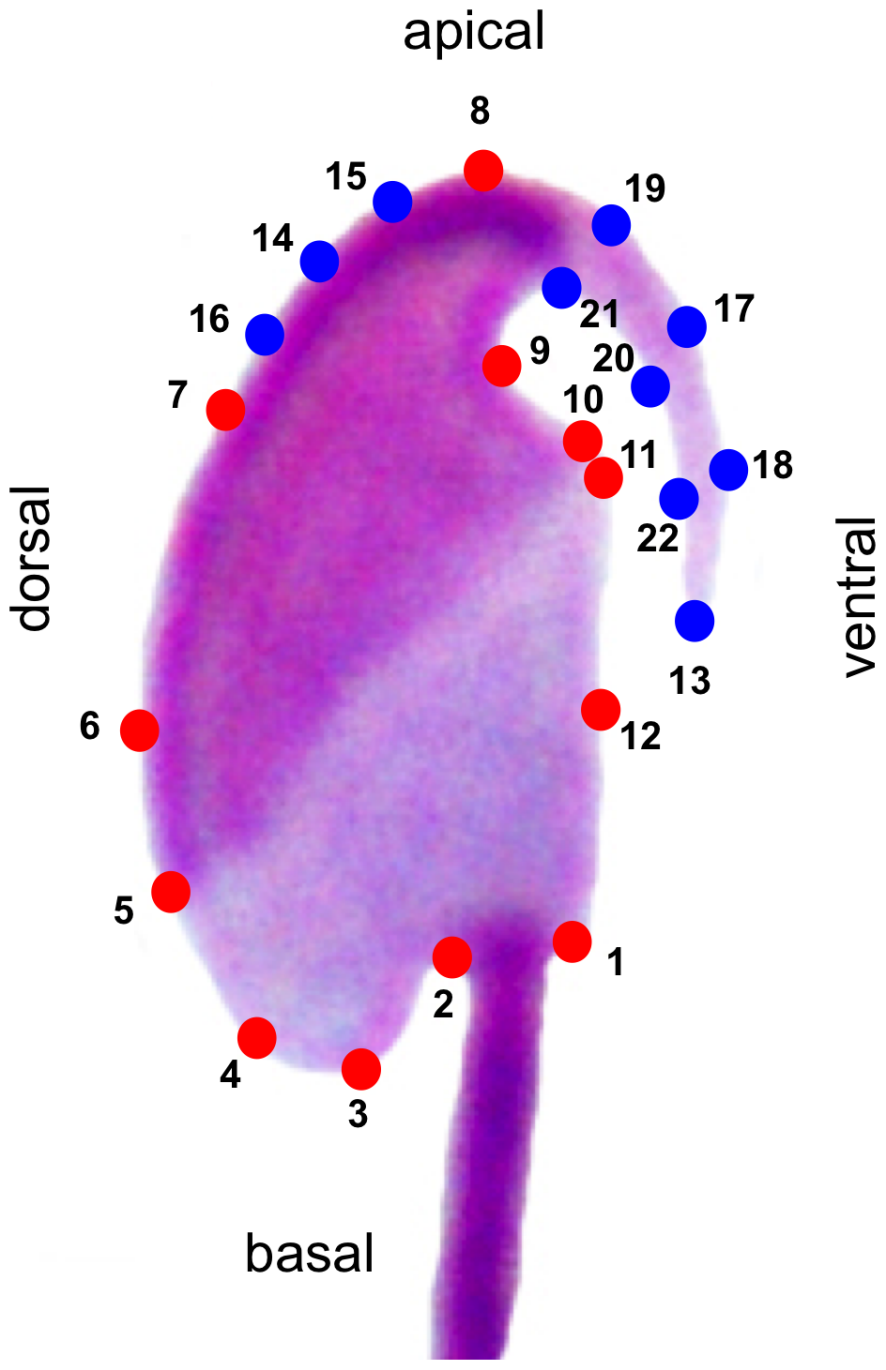
Landmark distribution on the sperm head. Red circles are landmarks whereas blue circles indicate semilandmarks. See description of landmarks in Table 1.

We chose 12 landmarks and 10 semilandmarks (Fig. 1, Table 1) distributed along the outlines of the sperm head, and which are characterized by relevant anatomical structures. Landmarks were digitized with TPSdig 2 (James Rohlf, Department of Ecology and Evolution, Stony Brook University, New York, USA) to get landmark coordinates. These coordinates were processed (reflected) with the Morpheus et al. software (Dennis Slice, Wake Forest University, Winston-Salem, North Carolina, USA) to correct the orientation in all sperm heads.

**Table 1:** Landmarks and semilandmarks used to asses sperm head morphology.

Landmark	Description	Type	
1	Flagellum insertion point, on the ventral side of the posterior ring	I	
2	Flagellum insertion point, on the dorsal side of the posterior ring	I	
3	Point of maximum length in head main axis (basal position)	I	
4	Beginning of the head curvature in the postacrosomal region	I	
5	Basal limit of the equatorial region of the acrosomal cap	I	
6	Maximum head width on the dorsal side of sperm	I	
7	Point of insertion of the basal end of the hook in the dorsal side of the head	I	
8	Point of maximum length in head main axis (apical position)	I	
9	Point of insertion of the basal end of the hook in the ventral side of the head	I	
10	Point of inflexion of the ventral side of the head	I	
11	Apical limit of the equatorial region of the acrosomal cap	I	
12	Maximun head width on the ventral side of sperm	I	
13	Tip of the hook	semilandmark	
14	Point at half of the distance between landmarks 7 and 8	semilandmark	
15	Point at half of the distance between landmarks 7 and 14	semilandmark	
16	Point at half of the distance between landmarks 8 and 14	semilandmark	
17	Point at half of the distance between landmarks 8 and 13	semilandmark	
18	Point at half of the distance between landmarks 17 and 13	semilandmark	
19	Point at half of the distance between landmarks 8 and 17	semilandmark	
20	Point at half of the distance between landmarks 9 and 13	semilandmark	
21	Point at half of the distance between landmarks 9 and 20	semilandmark	
22	Point at half of the distance between landmarks 13 and 20	semilandmark	

We then used Relwarp (James Rohlf, Department of Ecology and Evolution, Stony Brook University, New York, USA) for a generalized least squares (Procrustes) superimposition of the entire landmark configurations. During the Procrustes fit, the semilandmarks were slid so as to minimize the bending energy in a thin plates spline (TPS) between the Procrustes average (mean shape) and each of the individual specimens. Relwarp was also used to extract the centroid size values. Centroid size is defined as the square root of the squared, summed distances between all the landmarks and their center of gravity (centroid). It has been shown that, in absence of allometry, centroid size is the only measurement that is unrelated to shape [40].

### Statistical Analyses

All the statistical analyses were conducted on the slid shape coordinate data with MorphoJ [53] and Statistica v 6.0 (Statsoft, Tulsa, Oklahoma, USA). To assess intra-observer error, we measured landmarks on several spermatozoa repeatedly on five occasions (without semilandmarks) and performed a Procrustes ANOVA [54], [55]. The results showed that the variance due to landmark digitization is lower than the variance explained by shape differences between individuals (Table S1). Then, we assume that measurement error is negligible.

### Protocol for Comparison of Geometric Morphometrics Methods

Earlier studies used geometric morphometrics to analyze sperm shape variation in one mouse species, focusing mainly on differences in the shape of the hook [46], [47]. Here, we introduce a formal criterion for a rigorous comparison of geometric morphometrics methods with more traditional approaches assessing sperm head shape. This protocol consists of three steps:

(1) *Traditional analysis*: We quantified four head measures: length, width, area and perimeter. We also calculated four dimensions-derived parameters, namely, ellipticity  =  HL/HW, elongation  =  (L–W)/(L+W), regularity  =  πLW/4A, and roughness (also known as rugosity)  =  4πA/P^2^. This latter formula is the inverse of an earlier formula known as perimeter to area, P2A [56]. Analyses were carried out employing traditional ANOVA for mean shape differences between animals of different species.

(2) *Standardization:* In this step we follow Benazzi et al. [57] and standardize our shape data by a traditional variable (e.g., head length) so that after standardization no more variation of the traditional variable is present in the data. This standardization is achieved using a multivariate regression model of shape on the variable. The general equation for this model is (Y_1_, Y_2_…Y_n_)  =  (m_1_, m_2_…m_n_)X + (b_1_,b_2_…b_n_) + (r_1_,r_2_…r_n_), were Y are the shape variables (44 Procrustes shape coordinates), X is the variable used for standardization, m, b and r are vectors of slope, intercept coefficients and residuals respectively. During this step we produce shape data, which only contain residual variation, unrelated to the traditional variable (which is identical now in all cells after applying the regression model).

(3) *Geometric morphometrics analysis of standardization residuals*: Finally, we use Jonke et al. [58] protocol and compare the statistical results of both the traditional and the geometric morphometrics analysis. However, due to the regression in step 2, any group differences in the traditional variables measured have been removed, although other (residual) shape differences remain. Thus, if geometric morphometrics methods still detect significant shape differences (in residual shape data), then this method demonstrates a higher analytical morphometric resolution.

### Comparison Between Geometric Morphometrics Variables and Linear Dimensions

We regressed shape variables based on 22 landmarks (Table 1, Fig. 1) on the linear variables that are traditionally used as the main sperm head descriptors: length, width and area. These latter variables quantify size, so we added the centroid size parameter as the size measure used in geometric morphometrics, and conducted a correlation test to examine the degree of linear association between variables. We carried out an ANOVA, with Bonferroni post-hoc tests, to examine whether samples differed in their means.

In order to quantify if shape variation remains after cell standardization by multivariate regression of shape on linear variables, we calculated the Procrustes distances of the regression residuals between all species and their means.

### Comparison Between Geometric Morphometrics Variables and Dimension-Derived Parameters

This analysis was carried out to assess if dimensions-derived parameters are a worthy approximation to shape and, to a lesser extent, to size analysis. We performed an ANOVA with Bonferroni post-hoc tests, correlation and regression analyses between size and shape data and the dimensions-derived parameters ellipticity, elongation, regularity and roughness.

## Results

### Comparison Between Geometric Morphometrics Variables and Linear Dimensions

Linear dimensions of sperm heads differed between species (Table S2). Overall, head length showed a range of 2.05 µm (6.43 to 8.48 µm), head width range was 1.37 µm (2.93 to 4.31 µm) whereas head area exhibited a range of 8.29 µm^2^ (17.44 to 25.73 µm^2^). A one-way ANOVA of linear dimensions and centroid size revealed significant differences between all the variables except for head width. Bonferroni post-hoc tests also revealed differences in sperm head dimensions between species (Table S3).

We compared the information gathered by measurements of linear dimensions with shape analysis using geometric morphometrics. Regression analysis between Procrustes shape coordinates and linear dimensions showed significant relations for head length, head area and centroid size (Table 2). Head area explained 17.72% of total variance (*P*<0.001), centroid size explained 7.47% (*P*<0.001) whereas head length explained 4.23% (*P*<0.001). On the other hand, head width, which only explained 0.96% of total variance, showed no statistically significant relation (*P*  =  0.437). Thus, significant relations between some linear dimensions and shape coordinates were found, but differences in shape explained by dimensions were limited.

**Table 2:** Regression analyses between Procrustes shape coordinates and linear dimensions (1000 permutations).

Variable	% predicted	*P*
Head length	4.23	<0.001
Head width	0.96	0.437
Area	17.72	<0.001
Centroid size	7.47	<0.001

**Figure 2:**
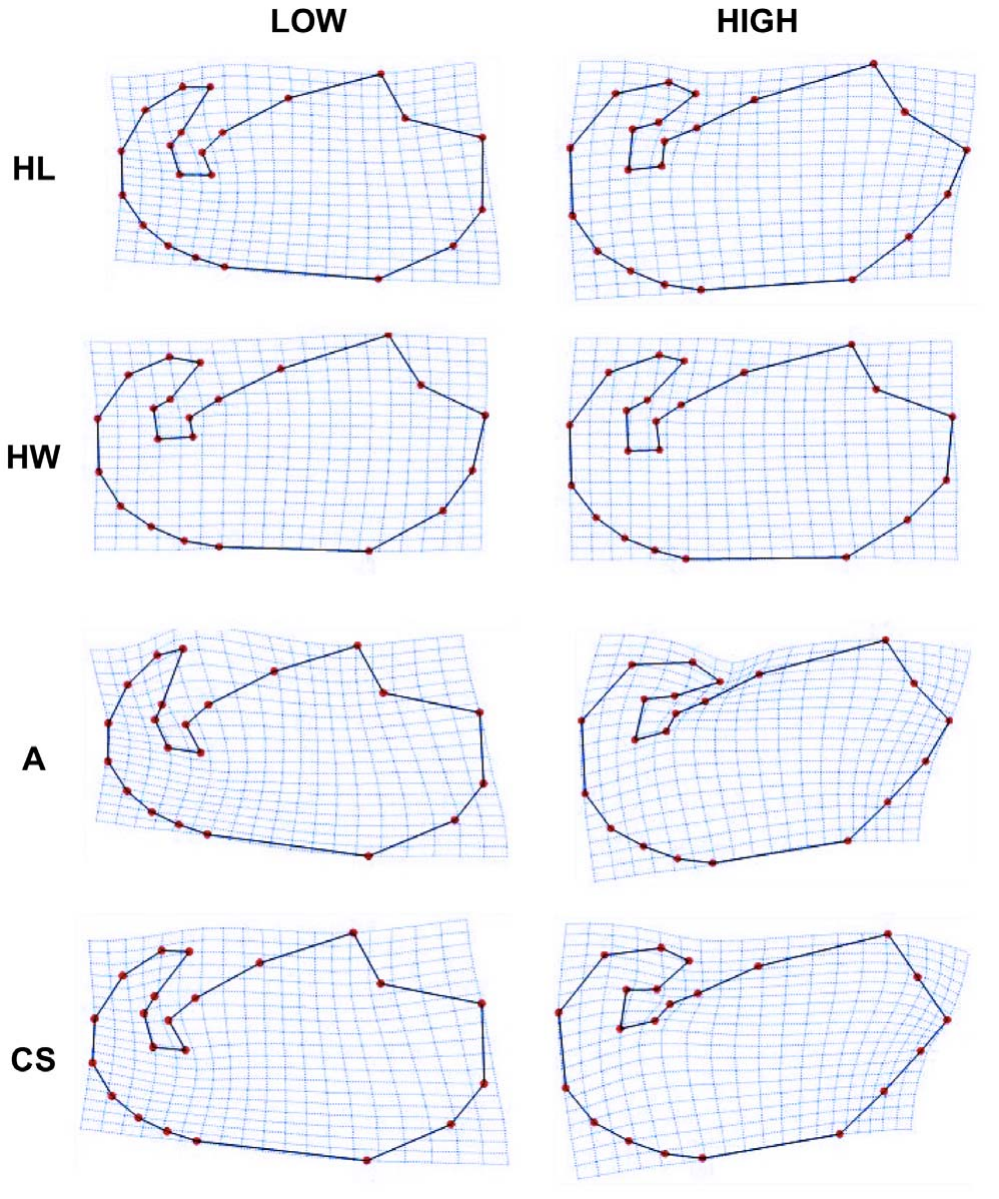
Shape changes due to head length (HL), head width (HW), area (A), and centroid size (CS). All shapes correspond to regression estimates at the minimum (LOW) and maximum (HIGH) of the actually observed, measured values.

With regards to shape differences due to changes in head dimensions, in longer heads the hook was more folded and the flagellum was inserted in a more basal position in comparison to shorter heads (Fig. 2, *first row, low vs high HL*). The dorsal curvature defined by landmarks 4, 5 and 6 was more flattened in longer heads, and the ventral outline through landmarks 10, 11, 12 and 1 tended to be straight (Fig. 2, *first row, low vs high HL*). These shape differences were also observed with changes in head area and in centroid size (Fig. 2, *third and fourth rows, low vs high HA or CS*). Head area and centroid size changes were also associated with key differences in the point of inflexion in the ventral side of the head (as defined by landmark 10) (Fig. 2, *third and fourth rows, low vs high HA or CS*). These differences seemed to be less prominent in long sperm heads. No clear shape differences were associated with differences in head width (Fig. 2, *second row, low vs high HW*). In summary, with low values of head length, head area, and centroid size, spermatozoa showed a rounder head shape, which tended to become more elongated as head length, area and centroid size values became higher.

To test for potential remaining shape differences after standardization with different head dimensions, we analyzed the mean Procrustes distances between the landmark configuration of species shape averages (Table 3). We found that species mean head shapes were different after being standardized to head length, head width, head area or centroid size. This indicates that, as expected, there are differences in shape that are not accounted for by differences in head dimensions.

**Table 3:** Procrustes distances between mean shapes of species after standardization to common linear dimensions (significant at *P*<0.0001).

	AS	AT	CG
Common head length			
AT	0.0806		
CG	0.1783	0.1265	
MA	0.1536	0.1183	0.1136
Common head width			
AT	0.0862		
CG	0.1798	0.1651	
MA	0.1510	0.1455	0.1146
Common area			
AT	0.1016		
CG	0.1392	0.0509	
MA	0.1416	0.0850	0.1103
Common centroid size			
AT	0.0936		
CG	0.1581	0.1047	
MA	0.1448	0.1196	0.1077

AS, Arvicola sapidus; AT, Arvicola terrestris; CG, Clethrionomys glareolus; MA, Microtus arvalis.

**Figure 3:**
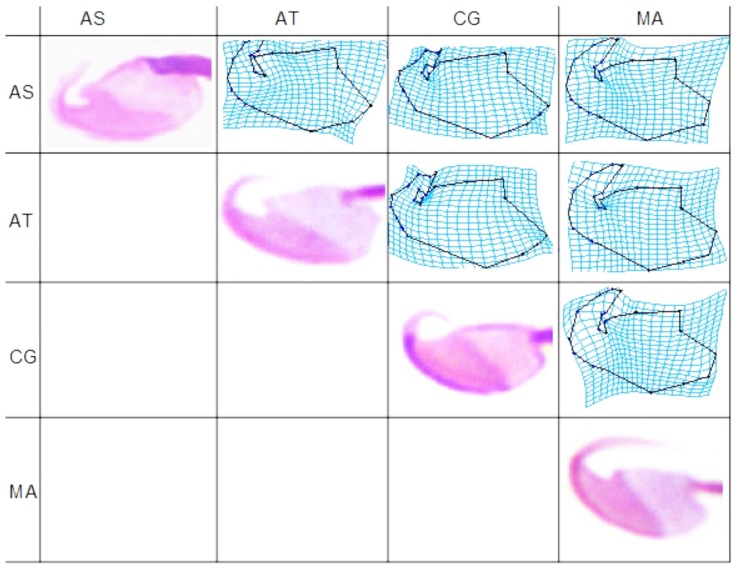
Sperm head mean deformation patterns. TPS deformation grids illustrate mean shape differences by deforming one species average into that of another species. AS, *Arvicola sapidus*; AT, *Arvicola terrestris*; CG, *Clethrionomys glareolus* and MA, *Microtus arvalis*.

Finally, we constructed a deformation matrix that allows for visualization of head shape differences between the mean shapes of each species (Fig. 3). We observed that major differences between species were present at the insertion point of the flagellum, the point of inflexion of the ventral side of the head (defined by landmark 10), the area of dorsal curvature defined by landmarks 4, 5 and 6, and hook shape and curvature. These deformation patterns coincided with those due to low and high values of head length, head width, head area and centroid size (cf. Fig. 2).

### Comparison Between Geometric Morphometrics Variables and Dimensions-Derived Parameters

These analyses compared two different approaches used to assess sperm head shape: the calculation of dimensions-derived parameters using various formulae *vs* Procrustes coordinates. Dimensions-derived parameters include ellipticity and elongation (which calculate, in two different ways, a ratio that measures length in relation to width of the sperm head), regularity (which approximates the sperm head perimeter to an ellipse), and roughness (which varies with a range between 0 and 1, and expresses the degree of resemblance of the sperm head to a circle).

The highest range of variation for dimensions-derived parameters among species was found for roughness  =  11.628 (10.434 to 13.633). The other parameters exhibited lower variation: regularity  =  0.975 (0.791 to 1.221), ellipticity  =  1.939 (1.616 to 2.339) and elongation  =  0.317 (0.235 to 0.401) (Table S4). An ANOVA of dimensions-derived parameters showed statistical differences between variables with the exception of ellipticity. Bonferroni post-hoc tests revealed differences in sperm head dimensions-derived parameters between species (Table S5).

Dimensions-derived parameters were compared with shape data obtained using geometric morphometrics. Regression analysis between dimensions-derived parameters and Procrustes coordinates revealed that regularity accounted for 15.4% of total variance (*P*<0.001), roughness explained 4.6% of variance (*P*  =  0.001), whereas ellipticity and elongation showed no statistically significant relation explaining, respectively, only 1.6% (*P*  =  0.135) and 1.8%, (*P*  =  0.095) of variance (Table 4). These results indicate that only two dimensions-derived parameters (regularity and roughness) did show some relationship with shape coordinates but that such relations were weak.

**Table 4:** Regression analyses between shape Procrustes and dimensions-derived parameters (1000 permutations).

Factor	% predicted	*P*
Ellipticity	1.6	0.135
Elongation	1.8	0.095
Regularity	15.4	<0.001
Roughness	4.6	0.001

**Figure 4:**
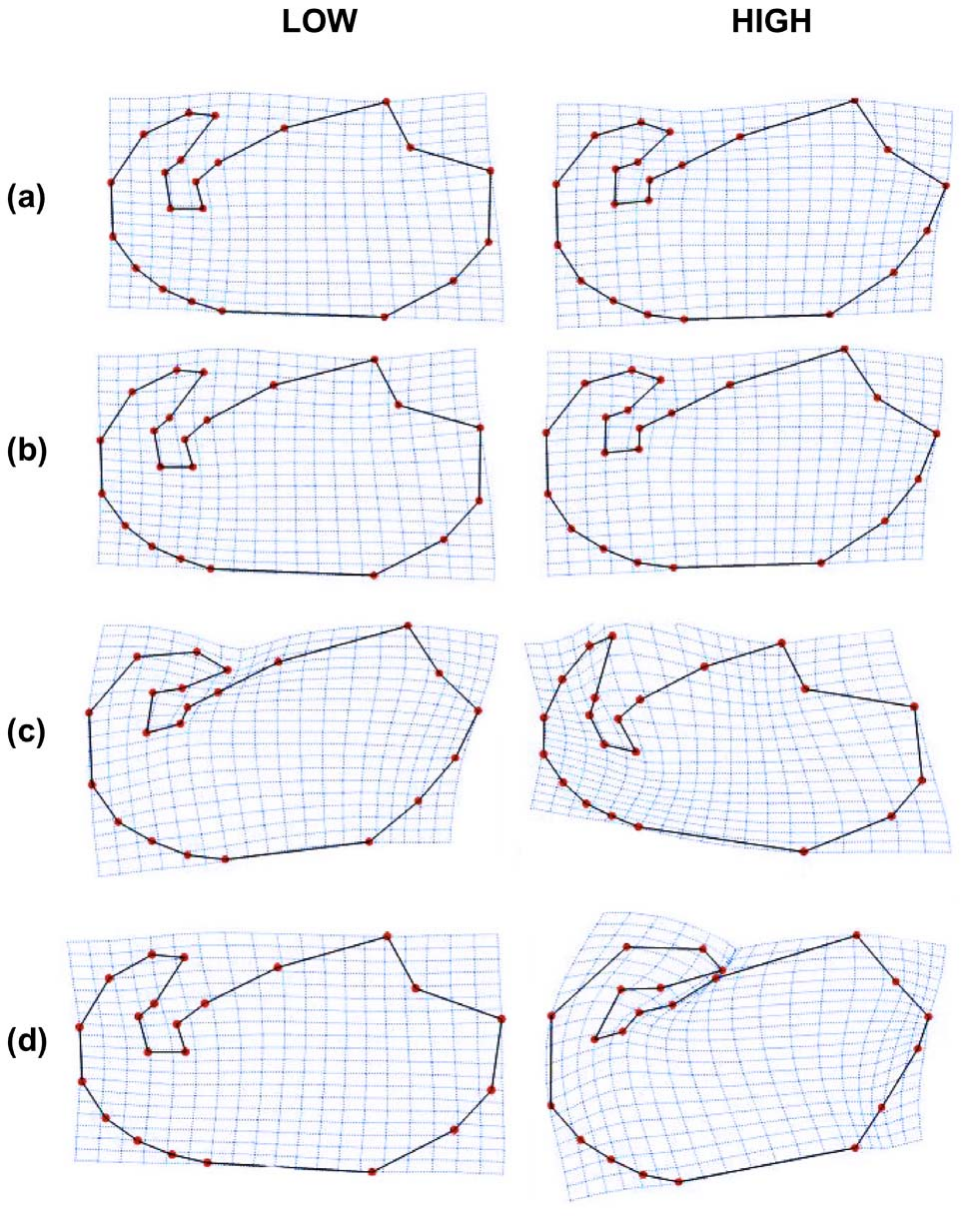
Shape changes due to (a) ellipticity; ( b) elongation; (c) regularity; (d) roughness. All shapes correspond to regression estimates at the minimum (LOW) and maximum (HIGH) of the actually observed values of the given parameters. Note that shape changes associated to regression models (a) and (b) are not statistically significant.

We examined differences in shape in relation to changes in dimensions-derived parameters. When shape was regressed on regularity the pattern observed was opposite to that seen with the other parameters (Fig. 4). At high values of regularity, (i.e., sperm head shape was rounder), the flagellum was inserted higher in the ventral aspect of the cell and the hook was not folded (Fig. 4, *third row*). At low values of regularity, this trend was reversed: the hook was folded, the flagellum was inserted in a more basal position (in the base of the head) and the cell shape looked thinner. On the other hand, at high values of ellipticity, elongation or roughness the flagellum was inserted in a more basal position, the hook was more folded, and the cell was slightly thinner than with low values of these dimensions-derived parameters (Fig. 4, *first, second and fourth rows*).

The analysis of mean Procrustes distances revealed that after standardization by ellipticity, elongation, regularity and roughness there were still differences in shape (Table 5). This indicates that shape differences clearly remain that are not accounted for by such dimensions-derived parameters.

**Table 5:** Procrustes distances between mean shapes of species after standardization to common values of dimensions-derived parameters ellipticity, elongation, regularity, and roughness (significant at *P*<0.0001, except where stated).

	AS	AT	CG
Common Ellipticity			
AT	0.0871		
CG	0.1779	0.1500	
MA	0.1520	0.1392	0.1129
Common Elongation			
AT	0.0869		
CG	0.1777	0.1487	
MA	0.1519	0.1380	0.1130
Common Regularity			
AT	0.0945		
CG	0.1325	0.0633 (*P* = 0.010)	
MA	0.1519	0.0989	0.1079
Common Roughness			
AT	0.0809		
CG	0.1775	0.1477	
MA	0.1543	0.1329	0.1173

AS, Arvicola sapidus; AT, Arvicola terrestris; CG, Clethrionomys glareolus; MA, Microtus arvalis.

**Figure 5:**
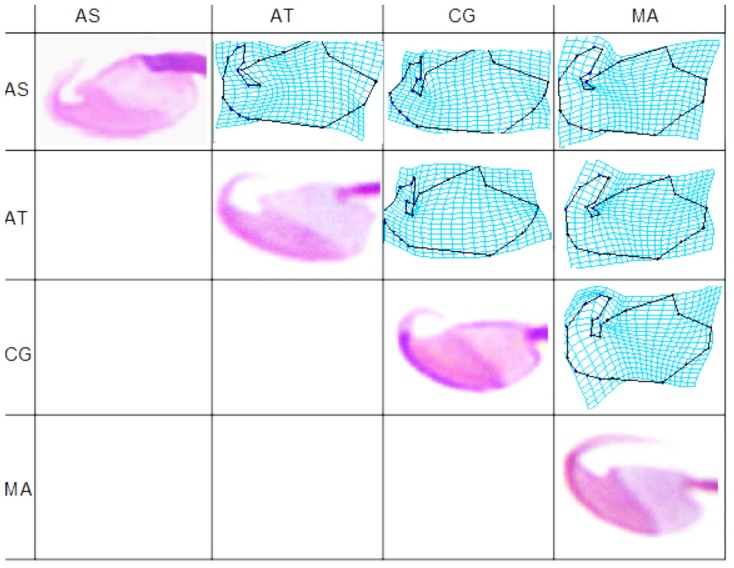
Sperm head mean deformation patterns after standardization to common regularity. TPS deformation grids illustrate mean shape differences by deforming one species average into that of another species. AS, *Arvicola sapidus*; AT, *Arvicola terrestris*; CG, *Clethrionomys glareolus* and MA, *Microtus arvalis*.

We examined mean shape differences after standardization to common values of regularity (Fig. 5); regularity was chosen for this analysis because it was the dimensions-derived parameter that better described shape (although its relation with shape coordinates was limited; see above). Shape differences between species (which were statistically significant) were mainly related to four regions: the point of inflexion of the ventral side of the head (defined by landmark 10), the area of dorsal curvature defined by landmarks 4, 5 and 6, the insertion point of the flagellum, and hook shape and curvature.

## Discussion

This study showed that geometric morphometrics offers a useful toolkit for sophisticated analysis of sperm head morphology. This approach may be valuable for the analysis of evolutionary variation between closely related species, phenotyping of rodent strains arising through genetic modification, or characterization of changes in sperm head shape that result from the action of reprotoxicants.

There may be considerable interspecific differences in sperm head shape. Closely related species may differ substantially in sperm head morphology [20], [21], [59], and differences in sperm head shape can aid in the identification of cryptic species [60], [61]. Changes in sperm head morphology may arise as a result of genetic alterations, as seen in mice with Y chromosome deletions [62], mutations (e.g., *azh*: [63], [64]) or genetic manipulation (e.g., [65]–[67]). In addition, changes in sperm head morphology may take place as a result of the action of chemical agents [68]. As a consequence, a "sperm morphology test" has been developed to identify chemicals that induce spermatogenic dysfunction and to assess their carcinogenic potential [69]. To aid in the evaluation of the impact of genetic alterations or toxic effects, classifications of sperm head abnormalities have been proposed [70], [71]. These classifications usually identify different categories of grossly misshapen sperm and, although sometimes a "quasi-normal" morph is recognized, this too may depart substantially from the normal sperm shape. The possibility of identifying subtle departures from a normal sperm head morphology may allow for a greater sensitivity in tests focusing on the impact of genetic alteration or the effect of reprotoxicants.

The landmarks identified and used in this study cover a broad range of structures in the sperm head that are susceptible to vary. Landmarks 1 and 2 define the site of flagellum insertion. Pairs of landmarks 3–8 and 6–12 define head length and head width, respectively. Landmarks 4 and 10 define two opposite-placed structures which vary analogously: they are prominent and rounded in some species and more flattened in others. Landmarks 5 and 11 define the boundary between the acrosomal and the post-acrosomal regions. The function of the rest of the landmarks and semilandmarks is to define the shape of the hook. We employed semilandmarks as a useful tool to analyze variation in hook shape, which is one of the structures experiencing more variation among rodent sperm. The hook plays an important role during sperm transport leading to fertilization, allowing the sperm cell to attach to the walls of the oviductal isthmus [72], [73]. The apical hook shows considerable morphological differences among species, which may have originated as a result of sperm competition [2], [46], [47], [74]. Studies in the house mouse using geometric morphometrics have in fact found differences in "hookedness" between different mouse lines under experimental selection or between subpopulations [46], [47]. Interestingly these studies used only four landmarks corresponding with key sperm structures, the remaining ones, including those defining the hook, being sliding semilandmarks and this may have reduced the sensitivity of analyses to detect variation in other head structures.

Many comparative or evolutionary studies rely on manual measurements of individual sperm cells using image-analysis software ([12], [13] and references therein). With the aim of making more objective and faster measurements of a larger number of cells, automated sperm morphometry analysis systems (ASMA) were introduced, with different systems now commercially available. Although there is currently a widespread use of this technology for human and domestic animal spermatozoa, whose sperm heads are usually round or paddle-shaped, ASMA tends to perform worse when sperm heads are less regular in shape. In any case, ASMA always relies on measurements of linear dimensions. Thus, regardless of whether manual or ASMA methods are used, measurements most commonly employed to analyze sperm head include length, width and area [1], [2], [12], [13], [49]. Additionally, ratios between these measures (head length/head width) are sometimes calculated and they are used to further discriminate sperm types mainly at the intraspecific level [17]. It is important to bear in mind that all these parameters are essentially size descriptors and, thus, unable to describe variation in spatial features of morphological variation (i.e., shape) with potential biological significance. The results of our study underscore that head length, head width and area are poor shape descriptors.

Removing the effect of size on sperm head differences, we managed to observe that differences could be identified in several anatomical features of the sperm head: the site of flagellum insertion, the dorsal and ventral curvatures defined by landmarks 4 and 10, respectively, and the shape of the hook. Spermatozoa with low values of head length have a rounder head shape that becomes elongated as head length increases. On the other hand, we did not observe shape differences related to head width. Sperm heads from many species have identical linear dimensions but considerable differences in shape, a situation we simulated statistically via standardization using multivariate regression and subsequent residual analysis. We found that some areas in the sperm head are very susceptible to change and that these changes actually occur in regions with little influence on linear dimensions. Therefore, using only linear dimensions in sperm analyses seriously limits the understanding of the complexity of these cells, something that is particularly important in rodents which exhibit a wide array of sperm head shapes. Shape analysis can thus reveal variation in biologically meaningful traits which are highly distinctive between sperm across taxa. A better characterization of sperm shape will lead to an improvement of our understanding of sperm biomechanics and hydrodynamic efficiency.

We also asked how ellipticity, elongation, regularity and roughness (which are calculated from linear dimensions) compared with Procrustes coordinates as shape descriptors. Our results showed that ellipticity and elongation are in fact two parameters that describe the same phenomenon: the ratio between sperm head lengthening and widening, thus providing what may be regarded as redundant information. In some studies, both are reported and results are treated as two different shape-like descriptors [27], [75]. Regularity, which measures how different spermatozoa are in shape from an ellipse, was found to be the parameter that explained the larger, although limited, amount of shape differences. This is due to the fact that sperm from muroid rodents resemble more an ellipse (because of the presence of the apical hook and the curvature in the dorso-basal region; landmark 4). Roughness measures the sperm shape variation between a circle and an ellipse. Rodent sperm examined in this study are elliptical or pyriform, so the descriptive value of this formula is limited. Our findings suggest that results obtained by geometric morphometrics analysis are able to explain a greater amount of shape variation than the dimensions-derived parameters, at least for rodent spermatozoa. ASMA has been originally developed for simple-shaped spermatozoa such as those from ungulates and primates, including humans. ASMA estimates sperm shape from a set of formulae approximating it to geometric figures that resemble the sperm outline. This approach is too simplistic to assess shape in complex spermatozoa such as those of rodents.

In conclusion, geometric morphometrics, as developed in this study, brings three main advantages in sperm morphology analyses: (a) it allows the assessment of size and shape separately, removing the size effect from shape, (b) it shows where the main shape changes occur in the sperm head, and (c) it provides an accurate method of quantifying shape and its use is not constrained by cell morphology. We believe the use of geometric morphometrics in sperm assessments offers an important new tool for both basic and applied studies.

## Supporting Information

Table S1 Measurement error assessment with Procrustes ANOVA for centroid size and shape coordinates.
(DOC)

Table S2 Descriptive statistics for linear dimensions in *Arvicola sapidus, Arvicola terrestris*, *Clethrionomys glareolus* and *Microtus arvalis*.
(DOC)

Table S3 One-way ANOVA and Bonferroni post-hoc tests for head length, head width and head area measured as linear dimensions describing size and centroid size. (a) One-way ANOVA, (b) Bonferroni post-hoc tests. Values (α) in bold are statistically significant (*P*<0.05). AS, *Arvicola sapidus*; AT, *Arvicola terrestris*; CG, *Clethrionomys glareolus*; MA, *Microtus arvalis*.
(DOC)

Table S4 Descriptive statistics for dimensions-derived parameters of sperm head morphology in *Arvicola sapidus, Arvicola terrestris*, *Clethrionomys glareolus* and *Microtus arvalis*.
(DOC)

Table S5 One-way ANOVA and Bonferroni post-hoc tests for dimensions-derived parameters used to assess sperm morphology. (a) One-way ANOVA, (b) Bonferroni post-hoc tests. Values (α) in bold are statistically significant (*P*<0.05). AS, *Arvicola sapidus*; AT, *Arvicola terrestris*; CG, *Clethrionomys glareolus*; MA, *Microtus arvalis*.
(DOC)
