# Supplementary material for: Geometric Morphometrics of Rodent Sperm Head Shape
Source: PLoS One. 2013 Nov 28;8(11):e80607. doi: 10.1371/journal.pone.0080607 (PMC3842927; doi:10.1371/journal.pone.0080607)
Supplement: Table S1 — Measurement error assessment with Procrustes ANOVA for centroid size and shape coordinates. (DOC) [file pone.0080607.s001.doc]

**Supplementary Table S1.** Measurement error assessment with Procrustes ANOVA for centroid size and shape coordinates.

(a) Centroid size

|  | SS | Degrees of freedom | MS | *F* | *P* |
| --- | --- | --- | --- | --- | --- |
| Individual | 10.430 | 15 | 0.695 | 5.98 | <0.001 |
| Error | 7.436 | 64 | 0.116 |  |  |

(b) Shape coordinates

|  | SS | Degrees of freedom | MS | *F* | *P* |
| --- | --- | --- | --- | --- | --- |
| Individual | 0.945 | 600 | 0.001 | 5.56 | <0.001 |
| Error | 0.725 | 2560 | 0.000 |  |  |
