# Supplementary material for: Geometric Morphometrics of Rodent Sperm Head Shape
Source: PLoS One. 2013 Nov 28;8(11):e80607. doi: 10.1371/journal.pone.0080607 (PMC3842927; doi:10.1371/journal.pone.0080607)
Supplement: Table S2 — Descriptive statistics for linear dimensions in Arvicola sapidus, Arvicola terrestris, Clethrionomys glareolus and Microtus arvalis. (DOC) [file pone.0080607.s002.doc]

**Supplementary Table S2.** Descriptive statistics for linear dimensions in *Arvicola sapidus,* *Arvicola terrestris*, *Clethrionomys glareolus* and *Microtus arvali* (N = 25/species).

|  | Mean | Minimum | Maximum | SD |
| --- | --- | --- | --- | --- |
| *Arvicola sapidus* |  |  |  |  |
| Head length | 7.104 | 6.521 | 7.719 | 0.299 |
| Head width | 3.958 | 3.679 | 4.306 | 0.213 |
| Area | 20.455 | 19.050 | 21.883 | 0.853 |
| Centroid size | 11.633 | 11.164 | 12.099 | 0.250 |
|  |  |  |  |  |
| *Arvicola terrestris* |  |  |  |  |
| Head length | 6.634 | 6.208 | 7.053 | 0.228 |
| Head width | 3.607 | 3.195 | 4.090 | 0.215 |
| Area | 17.627 | 15.890 | 19.594 | 1.006 |
| Centroid size | 11.139 | 10.434 | 11.895 | 0.338 |
|  |  |  |  |  |
| *Clethrionomys glareolus* |  |  |  |  |
| Head length | 7.138 | 6.767 | 7.493 | 0.200 |
| Head width | 3.593 | 3.101 | 3.931 | 0.198 |
| Area | 22.691 | 21.342 | 25.510 | 0.898 |
| Centroid size | 11.972 | 11.547 | 13.633 | 0.446 |
|  |  |  |  |  |
| *Microtus arvalis* |  |  |  |  |
| Head length | 6.961 | 6.564 | 7.491 | 0.308 |
| Head width | 3.259 | 2.791 | 3.689 | 0.231 |
| Area | 20.318 | 18.063 | 22.565 | 1.273 |
| Centroid size | 11.768 | 11.111 | 12.592 | 0.328 |
|  |  |  |  |  |
| *All species* |  |  |  |  |
| Head length | 7.226 | 6.430 | 8.481 | 0.355 |
| Head width | 3.627 | 2.934 | 4.306 | 0.292 |
| Area | 21.070 | 17.438 | 25.728 | 1.768 |
| Centroid Size | 11.628 | 10.434 | 13.633 | 0.461 |
