# Supplementary material for: Geometric Morphometrics of Rodent Sperm Head Shape
Source: PLoS One. 2013 Nov 28;8(11):e80607. doi: 10.1371/journal.pone.0080607 (PMC3842927; doi:10.1371/journal.pone.0080607)
Supplement: Table S3 — One-way ANOVA and Bonferroni post-hoc tests for head length, head width and head area measured as linear dimensions describing size and centroid size. (a) One-way ANOVA, (b) Bonferroni post-hoc tests. Values (α) in bold are statistically significant (P<0.05). AS, Arvicola sapidus; AT, Arvicola terrestris; CG, Clethrionomys glareolus; MA, Microtus arvalis. (DOC) [file pone.0080607.s003.doc]

**Supplementary Table S3.** One-way ANOVA and Bonferroni post-hoc tests for head length, head width and head area measured as linear dimensions describing size and centroid size. (a) One-way ANOVA, (b) Bonferroni post-hoc tests. Values (α) in bold are statistically significant (*P* < 0.05). AS, *Arvicola sapidus*; AT, *Arvicola terrestris*; CG, *Clethrionomys glareolus*; MA, *Microtus arvalis*.

(a)

|  | SS | Degrees of freedom | MS | *F* | *P* |
| --- | --- | --- | --- | --- | --- |
| Head length | 4.136 | 3 | 1.379 | 22.08 | 0.000 |
| Head width | 0.014 | 3 | 0.005 | 0.122 | 0.947 |
| Area | 332 | 3 | 110.7 | 81.6 | 0.000 |
| Centroid size | 9.423 | 3 | 3.141 | 25.91 | 0.000 |

(b)

| ***Head length*** | | | | |  | ***Head width*** | | | | |
| --- | --- | --- | --- | --- | --- | --- | --- | --- | --- | --- |
| species | AS | AT | CG | MA |  | species | AS | AT | CG | MA |
| AS |  | **0.000** | 1.000 | 1.000 |  | AS |  | 1.000 | 1.000 | 1.000 |
| AT |  |  | **0.000** | **0.000** |  | AT |  |  | 1.000 | 1.000 |
| CG |  |  |  | 1.000 |  | CG |  |  |  | 1.000 |
| MA |  |  |  |  |  | MA |  |  |  |  |

| ***Head area*** | | | | |  | ***Centroid size*** | | | | |
| --- | --- | --- | --- | --- | --- | --- | --- | --- | --- | --- |
| species | AS | AT | CG | MA |  | species | AS | AT | CG | MA |
| AS |  | **0.000** | **0.000** | 0.655 |  | AS |  | **0.000** | 0.005 | 1.000 |
| AT |  |  | **0.000** | **0.000** |  | AT |  |  | **0.000** | **0.000** |
| CG |  |  |  | **0.000** |  | CG |  |  |  | 0.244 |
| MA |  |  |  |  |  | MA |  |  |  |  |
