# Supplementary material for: Geometric Morphometrics of Rodent Sperm Head Shape
Source: PLoS One. 2013 Nov 28;8(11):e80607. doi: 10.1371/journal.pone.0080607 (PMC3842927; doi:10.1371/journal.pone.0080607)
Supplement: Table S4 — Descriptive statistics for dimensions-derived parameters of sperm head morphology in Arvicola sapidus, Arvicola terrestris, Clethrionomys glareolus and Microtus arvalis. (DOC) [file pone.0080607.s004.doc]

**Supplementary Table S4.** Descriptive statistics for dimensions-derived parameters of sperm head morphology in *Arvicola sapidus,* *Arvicola terrestris*, *Clethrionomys glareolus* and *Microtus arvalis* (N = 25/species).

|  | Mean | Minimum | Maximum | SD |
| --- | --- | --- | --- | --- |
| *Arvicola sapidus* |  |  |  |  |
| Ellipticity | 1.797 | 1.640 | 1.968 | 0.079 |
| Elongation | 0.284 | 0.242 | 0.326 | 0.020 |
| Regularity | 1.082 | 0.929 | 1.282 | 0.099 |
| Roughness | 11.633 | 11.164 | 12.099 | 0.250 |
| *Arvicola terrestris* |  |  |  |  |
| Ellipticity | 2.284 | 2.126 | 2.535 | 0.111 |
| Elongation | 0.390 | 0.360 | 0.434 | 0.020 |
| Regularity | 0.993 | 0.815 | 1.102 | 0.063 |
| Roughness | 11.139 | 10.434 | 11.895 | 0.338 |
| *Clethryonomys glareolus* |  |  |  |  |
| Ellipticity | 1.992 | 1.740 | 2.339 | 0.128 |
| Elongation | 0.330 | 0.270 | 0.401 | 0.028 |
| Regularity | 0.887 | 0.791 | 0.959 | 0.039 |
| Roughness | 11.972 | 11.547 | 13.633 | 0.446 |
| *Microtus arvalis* |  |  |  |  |
| Ellipticity | 1.944 | 1.679 | 2.280 | 0.124 |
| Elongation | 0.319 | 0.253 | 0.390 | 0.028 |
| Regularity | 0.958 | 0.865 | 1.050 | 0.049 |
| Roughness | 11.768 | 11.111 | 12.592 | 0.328 |
| *All species* |  |  |  |  |
| Ellipticity | 1.939 | 1.616 | 2.339 | 0.144 |
| Elongation | 0.317 | 0.235 | 0.401 | 0.033 |
| Regularity | 0.975 | 0.791 | 1.221 | 0.086 |
| Roughness | 11.628 | 10.434 | 13.633 | 0.461 |
