# Supplementary material for: Geometric Morphometrics of Rodent Sperm Head Shape
Source: PLoS One. 2013 Nov 28;8(11):e80607. doi: 10.1371/journal.pone.0080607 (PMC3842927; doi:10.1371/journal.pone.0080607)
Supplement: Table S5 — One-way ANOVA and Bonferroni post-hoc tests for dimensions-derived parameters used to assess sperm morphology. (a) One-way ANOVA, (b) Bonferroni post-hoc tests. Values (α) in bold are statistically significant (P<0.05). AS, Arvicola sapidus; AT, Arvicola terrestris; CG, Clethrionomys glareolus; MA, Microtus arvalis. (DOC) [file pone.0080607.s005.doc]

**Supplementary Table S5.** One-way ANOVA and Bonferroni post-hoc tests for dimensions-derived parameters used to assess sperm morphology. (a) One-way ANOVA, (b) Bonferroni post-hoc tests. Values (α) in bold are statistically significant (*P* < 0.05). AS, *Arvicola sapidus*; AT, *Arvicola terrestris*; CG, *Clethrionomys glareolus*; MA, *Microtus arvalis*.

**(a)**

|  | SS | Degrees of freedom | MS | *F* | *P* |
| --- | --- | --- | --- | --- | --- |
| Ellipticity | 0.314 | 3 | 0.105 | 5.745 | 0.011 |
| Elongation | 0.018 | 3 | 0.006 | 6.133 | 0.000 |
| Regularity | 0.417 | 3 | 0.139 | 40.82 | 0.000 |
| Roughness | 0.244 | 3 | 0.081 | 4.187 | 0.008 |

**(b)**

| ***Ellipticity*** | | | | |  | ***Elongation*** | | | | |
| --- | --- | --- | --- | --- | --- | --- | --- | --- | --- | --- |
| species | AS | AT | CG | MA |  | species | AS | AT | CG | MA |
| AS |  | **0.011** | 1.000 | 1.000 |  | AS |  | **0.008** | 1.000 | 1.000 |
| AT |  |  | **0.001** | **0.039** |  | AT |  |  | **0.008** | 0.025 |
| CG |  |  |  | 1.000 |  | CG |  |  |  | 1.000 |
| MA |  |  |  |  |  | MA |  |  |  |  |

| ***Regularity*** | | | | |  | ***Roughness*** | | | | |
| --- | --- | --- | --- | --- | --- | --- | --- | --- | --- | --- |
| species | AS | AT | CG | MA |  | species | AS | AT | CG | MA |
| AS |  | **0.000** | **0.000** | 0.388 |  | AS |  | 0.066 | 1.000 | 1.000 |
| AT |  |  | **0.000** | **0.000** |  | AT |  |  | **0.006** | 0.350 |
| CG |  |  |  | **0.000** |  | CG |  |  |  | 0.861 |
| MA |  |  |  |  |  | MA |  |  |  |  |
